# Different Clinical Relevance of Anti-Citrullinated Protein Antibodies in RA Patients

**DOI:** 10.1134/S160767292370031X

**Published:** 2023-10-13

**Authors:** A. S. Avdeeva, M. V. Cherkasova, E. L. Nasonov

**Affiliations:** 1 Nasonova Research Institute of Rheumatology, Moscow, Russia; 2 Department of Rheumatology, Institute of Professional Education, Sechenov First Moscow State Medical University (Sechenov University), Moscow, Russia

**Keywords:** rheumatoid arthritis, disease activity, bone destruction, anti-citrullinated proteins antibodies

## Abstract

The objective of the study was to find a potential relationship between ACPAs and disease activity, bone destruction, and ACPA responses to various therapeutic regimens. The study included 232 patients with rheumatoid arthritis (RA); 90 patients had early RA, and 142 patients had an advanced stage of the disease. 77 (85.6%) patients with early RA were highly positive for anti-CCP, and 29 (70.7%) patients were highly positive for anti-MCV. A positive correlation was found between anti-MCV and DAS28 (*r* = 0.4; *p* = 0.04). As for advanced RA, 78 (80.4%) patients were high-positive for anti-CCP, and 70 (79.5%) were high-positive for anti-MCV. There was a positive correlation between anti-MCV concentration and SDAI (*r* = 0.4; *p* = 0.02), as well as CDAI (*r* = 0.4; *p* = 0.02). No significant correlations were found between the anti-CCP levels and activity indices, anti-CCP and acute-phase parameters in both early and advanced RA groups. Higher total Sharp scores (96.5 (65.0–122.0)) were found in pts highl-positive for anti-MCV (*n* = 79), compared to low-positive/negative (*n* = 27) patients (57.0 (31.0–88.0); *p* < 0.05). Anti-MCV levels dropped significantly in pts on rituximab and tocilizumab therapy at weeks 12 and 24 after initiation of treatment, while high anti-CCP concentration persisted throughout the treatment. Anti-MCV levels correlated with inflammatory activity and development of bone destruction and decreased in pts on treatment. Anti-CCP was less responsive and showed minor changes during treatment; therefore, its thorough monitoring was not feasible.

Rheumatoid arthritis (RA) as an autoimmune disease is characterized by the presence in the blood serum and synovial fluid of a wide range of autoantibodies with different specificity, including rheumatoid factors (RFs) of classes IgM, IgA, and IgG, as well as autoantibodies, reacting with various antigenic epitopes. Their universal characteristic is posttranslational modification [1, 2], mediated by their citrullination, carbamylation, acetylation, peroxidation, etc. [3, 4]. The study of the level of anti-citrullinated protein antibodies (ACPAs) is important both for the diagnosis of RA and for assessing the severity and prognosis of the disease. ACPAs is a large group of antibodies, including the antibodies to citrullinated fibrinogen, antibodies to cyclic citrullinated peptide (anti-CCP), antibodies to modified citrullinated vimentin (anti-MCV), and antibodies to citrullinated α-enolase [5–9]. Currently, in the ACPA group, the determination of the content of anti-CCP and anti-MCV is most widely used in clinical practice. The determination of anti-CCP is very useful for diagnosing early RA, differential diagnosing with other rheumatic diseases, and for predicting severe erosive joint damage. Hyperproduction of anti-CCP (especially in combination with RF) is associated with the development of extra-articular (systemic) manifestations [10], the risk of overall mortality associated with the development of comorbid conditions (primarily cardiovascular complications [11, 12]), as well as with “resistance” or, conversely, “sensitivity” to therapy with disease-modifying antirheumatic drugs (DMARDs) and genetically engineered biological drugs (biologics) [13]. Anti-MCVs have a high sensitivity and detection efficiency for the diagnosis of RA but a lower specificity that anti-CCPs [8–14]. It was shown that anti-MCV may be a better predictor of unfavorable radiological prognosis of articular destruction than anti-CCP [14]. However, different authors provide conflicting data on the association between anti-MCV and disease activity.

In recent years, ample data have been accumulated indicating that ACPAs (and rheumatoid factors) are not only a sensitive and specific biomarker of RA (an “innocent bystander”) but may have pathogenetic significance, functioning as additional mediators of inflammation and bone tissue destruction. It is assumed that ACPAs, being a very heterogeneous population of autoantibodies, differ significantly in pathogenic potential and contribution to the development and progression of RA at different stages of the disease. This, in turn, depends on the impact of additional exogenous or endogenous factors (the so-called “second hit”), such as infection, genetic factors, the repertoire of T-cell receptors, epigenetic disorders, etc., which enhance the “pro-inflammatory” potential of autoantibodies. However, the true nature of these factors and the specific mechanisms leading to increased “pathogenicity” of autoantibodies requires further investigation [15].

Recently, the concept of “immunological remission” has been introduced into clinical practice [16]. Immunological remission is a condition characterized by the absence of clinical and instrumental signs of inflammation and confirmed IgM RF and/or anti-CCP seroconversion. Thus, taking into account the contribution of autoantibodies to the pathogenesis of RA, it is `important to assess their relationship with the activity of the disease, the development of destructive changes in the joints, the dynamics against the background of various treatment regimens, and to clarify the possibility of achieving immunological remission of RA.

The study included 232 patients diagnosed with RA, 187 patients were examined in dynamics. All patients were hospitalized and were under inpatient treatment at the Nasonova Research Institute of Rheumatology, Moscow, Russia. The general clinical and immunological characteristics of patients are presented in Table 1.

**Table 1.** Clinical and immunological characterization of patients included in the study

Parameter	Patients with early RA, *n* = 90	Patients with established RA, *n* = 142	Healthy donors, *n* = 60
Gender:			
–males	16	19	6
–females	74	123	54
Age, years Me [25–75 percentile]	53.0 (38.0–58.5)	51.0 (43.0–60.0)	42.3 (35.8–54.6)
RA duration time, months/Me [25–75 percentile]	5.0 (4.0–9.0)	56.0 (24.0–96.0)	–
Radiologic stage, *n* (%):			
–I	26 (28.9)	4 (2.8)	–
–II	59 (65.6)	54 (38.0)	
–III	5 (5.6)	61 (42.9)	
–IV	0	23 (16.2)	
Functional class, *n* (%):			
–I	37 (41.1)	22 (15.5)	–
–II	50 (55.6)	112 (78.9)	
–III	3 (3.3)	8 (5.6)	
–IV	0		
DAS 28, Me [25–75 percentile]	5.3 (4.4–6.1)	6.2 (5.5–6.8)	–
ESR, mm/h Me [25–75 percentile]	33.0 (18.0–50.0)	48.0 (30.0–70.0)	–
CRP, mg/l Me [25–75 percentile]	18.8 (4.2–48.5)	27.2 (13.8–49.0)	0.8 (0.2–1.7)
IgM RF positive, *n* (%)	72 (80)	116 (81.7)	0
Anti-CCP positive, *n* (%)	83 (92.2)	121 (85.2)	0

Among the patients included in the study, women predominated (the ratio of women and men in the group of patients with early RA was 4.6 to 1; with advanced RA, 6.5 to 1) of middle age (the median age in the group of patients with early RA was 53 years; with advanced RA, 51 years). The median duration of the disease in the group of patients with early RA was 5 months; with advanced RA, 56 months. The majority of patients had X-ray stage II and III, functional class II. Patients had elevated levels of acute-phase parameters, the majority of patients were positive for RF IgM (80% of patients from the group with early RA and 81.7% from the group with advanced RA) and for anti-CCP (92.2 and 85.2%, respectively).

Depending on the therapy, the patients were divided into a number of groups: patients with early RA did not receive therapy with DMARDs before being included in the study; dose escalation to 20–25 mg per week. All of them were initiated on methotrexate (MT) (Metoject) therapy (subcutaneously, 10 mg per week, with rapid dose escalation to 20–25 mg per week).

Every 12 weeks, the patients were examined by an expert and, depending on the activity of the disease, the issue of changing the treatment or continuing the previous therapy was decided. In the case of insufficient effectiveness of MT, biologics were added to the treatment.

In the group of patients with advanced RA, several subgroups were distinguished: patients receiving anti-B-cell therapy—34 patients received original rituximab (RTM) (12 patients (35%) at a dose of 500 mg and 22 patients (65%) at a dose of 1000 mg) intravenously with an interval of 2 weeks simultaneously with therapy with DMARDs, NSAIDs, and GCs, and 20 patients received therapy with the biosimilar RTM (Acellbia) at a dose of 600 mg intravenously according to the same scheme. Forty-three patients received therapy with monoclonal antibodies to IL-6 receptors, tocilizumab (TCZ) at a dose of 8 mg/kg intravenously with an interval of 4 weeks during therapy with DMARDs, NSAIDs, and GCs. The control group consisted of 60 healthy donors matched in sex and age with the examined patients.

Quantitative determination of anti-CCP in blood serum was performed by electrochemiluminescence technique on a Cobas e411 analyzer (Roche, Switzerland) (upper limit of normal 17.0 U/mL) and by ELISA using commercial kits of reagents (Axis-Shield, UK) (upper normal limit 5.0 U/mL). High-positive (>50.0 U/mL for the electrochemiluminescence technique and >15.0 U/mL for ELISA), low-positive (17.0–50.0 and 5.0–15.0 U/mL, respectively), and negative (≤17.0 and ≤5.0 U/mL, respectively) levels of anti-CCP. The concentration of anti-MCV in blood serum was determined by ELISA using a commercial reagent kits (ORGENTEC Diagnostika, Germany). According to the manufacturer’s recommendations, the upper normal limit for anti-MCV was 20 U/mL. The high positive (>60 U/mL), low-positive (20–60 U/mL), and negative (≤20 U/mL) levels of anti-MCV were detected. All patients underwentradiography of the hands and distal parts of the feet in the direct (anterior-posterior) projection using the  standard modes. The progression of the destructive process in the joints was assessed by the van der Heijde-modified Sharp score (1989) with the determination of the total erosion score and the total score of the radiological progression of the joints by the 12th month of observation.

The results were statistically processed using the Statistica 10.0 software package (StatSoft, United States), including the generally accepted methods of parametric and nonparametric analysis. For the parameters whose distribution differed from normal, the Mann–Whitney test was used when comparing two groups and the Kruskal–Wallace test was used when comparing three or more groups. The results are presented as a median (Me) with an interquartile range of 25–75 percentile. Correlation analysis was performed using the Spearman’s method. Differences were considered statistically significant at *p* < 0.05.

Among the patients with early RA, a high-positive level of anti-CCP was detected in 77 (85.6%), low positive in 6 (6.7%), and negative in 7 (7.8%) patients. The level of anti-MCV was determined in a group of 41 patients. A high-positive level of this parameter was recorded in 29 (70.7%) patients, a low positive level in 8 (19.5%) patients, and a negative level in 4 (9.8%) patients.

There was a positive correlation between anti-MCV and NPJ 28 (*r* = 0.4,  *p* = 0.004), NSJ 28 (*r* = 0.38,  *p* = 0.04) and DAS 28 (*r* = 0.4, *p* = 0.04). No significant correlation between anti-CCP levels and clinical and laboratory indices of inflammatory activity was found. There were no significant differences in the level of autoantibodies in the groups of patients depending on the activity of the disease.

Among the patients with advanced RA, a high positive level of anti-CCP was detected in 78 (80.4%), a low positive level in 8 (8.2%), and a negative level in 11 (11.3%) patients; a high positive level of anti-MCV was detected in 70 (79.5%) patients, a low positive level in 8 (9.1%) patients, and a negative level in 10 (11.4%) patients.

There was a positive correlation between the concentration of anti-MCV and NPJ 28 (*r* = 0.2, *p* = 0.04), SDAI (*r* = 0.4, *p* = 0.02), and CDAI (*r* = 0.4, *p* = 0.02). Significant correlations between the level of anti-CCP and activity indices and acute-phase parameters were not found.

To assess the association of autoantibody levels with the development of destructive changes in the joints, all patients were divided into groups depending on the level of positivity for anti-CCP and anti-MCV. No significant differences in articular destruction in the groups of patients depending on the positivity for anti-CCP were found (*p* > 0.05). Among the patients highly positive for anti-MCV (*n* = 79), there were more constrictions (82.0 (60.0–105.0)) and a higher Sharp total score (96.5 (65.0–122.0)) compared with the negative or low-positive patients (50.0 (29.0–82.0) and 57.0 (31.0–88.0), respectively; *n* = 27, *p* <0.05).

Then, we assessed the dynamics of anti-CCP and anti-MCV against the background of various RA treatment strategies.

**Dynamics of the level of autoantibodies against the background of anti-B-cell therapy**. Before the start of RTM therapy, among the patients treated with the original RTM, the levels of autoantibodies (Me; IR) did not differ significantly in the patients with good effect and with moderate or no effect of therapy (*p* > 0.05). In the group of patients receiving the biosimilar, among patients who responded well to treatment, the level of anti-MCV was significantly higher than in the patients with a satisfactory effect of treatment  or its absence (1000 (1000–1000) and 225.9 (60.8–654.5) U/mL, respectively; *p* < 0.05). The dynamics of laboratory biomarkers depending on the effect of the drug is presented in Tables 2, 3.

**Table 2.** Dynamics of the level of autoantibodies in the original RTM therapy group (*n* = 34), Me [25–75 percentile]

Parameter	Anti-CCP, U/mL	Anti-MCV, U/mL
Total group, *n* = 34		
–baseline	100.0 (37.9–100.0)	559.4 (139.2–1000.0)
–12 weeks	100.0 (26.1–100.0)	295.9 (74.0–962.7)^1^
–24 weeks	100.0 (29.0–100.0)	194.7 (58.3–844.8)^1^
Good response, *n* = 15		
–baseline	100.0 (37.9–100.0)	950.9 (139.2–1000.0)
–12 weeks	100.0 (100.0–100.0)	757.9 (113.9–1000.0)
–24 weeks	100.0 (27.2–100.0)	606.2 (64.6–988.8)
Moderate/no response, *n* = 19		
–baseline	100.0 (24.7–100.0)	298.7 (132–658.3)
–12 weeks	94.1 (26.1–100.0)	127.5 (58.3–418.5)^1^
–24 weeks	100.0 (14.6–100.0)	110.9 (54.9–449)^1^

^1^*p* < 0.05 compared to baseline.

**Table 3.** Dynamics of the level of autoantibodies in the RTM biosimilar group (*n* = 20), Me [25–75 percentile]

Parameter	Anti-CCP, U/mL	Anti-MCV, U/mL
Total group, *n* = 20		
–baseline	112.7 (18.3–264.8)	392.6 (75.7–1000.0)
–12 weeks	71.7 (12.4–161.6)	210.5 (40.3–940.6)^1^
–24 weeks	61.3 (13.12–129.4)	153.8 (43.1–702.8)
Good response, *n* = 5		
–baseline	71.2 (31.9–264.5)	1000.0 (1000.0–1000.0)
–12 weeks	71.6 (61.9–227.8)	1000.0 (475.1–1000.0)
–24 weeks	42.4 (13.3–53.2)	295.8 (132.5–329.8)^1^
Moderate/no response, *n* = 15		
–baseline	120.4 (14.2–265.1)	225.9 (60.8–654.5)^2^
–12 weeks	71.8 (12.2–154.9)	109.6 (22.9–415.9)
–24 weeks	69.6 (13–135.1)	126.9 (24.4–832.4)

^1^*p*< 0.05 compared to baseline;  ^2^*p* < 0.05 between groups with good response and moderate or no response.

The concentration of anti-CCP in the blood sera of patients who responded to therapy remained high throughout the entire duration of RTM therapy, both among the patients who received the original drug and the biosimilar. In 7% of anti-CCP-positive patients in the original drug group and 15% of patients in the biosimilar group seroconverted to anti-CCP with negative results. The level of anti-MCV significantly decreased by 38% and 62% 12 and 24 weeks after the start of RTM in the first group and by 46.4 and 60.8% in the second group, respectively (Tables 2, 3).

Taking into account the fact that the use of anti-B-cell therapy was accompanied by negative seroconversion, we estimated the percentage of patients who achieved immunological remission during treatment. By the 24th week of therapy, 4 (7.4%) patients achieved clinical remission and anti-CCP seroconversion.

**Dynamics of the level of autoantibodies against the background of therapy with monoclonal antibodies to IL-6 receptors.** The dynamics of laboratory biomarkers was assessed depending on the response to therapy according to the EULAR criteria and is presented in Table 4.

**Table 4.** Dynamics of the level of autoantibodies in the TCZ group, Me [25–75 percentile]

Parameter	Anti-CCP, U/mL	Anti-MCV, U/mL
Total group, *n* = 43		
–baseline	354.8 (67.9–500.0)	762.3 (106.9–2393.1)
–2 weeks	290.5 (65.6–413.6) ^1^	627.9 (116.6–1481.6)
–4 weeks	388.9 (83.7–500.0)	312.2 (81.2–925.3) ^1^
–8 weeks	500.0 (84.4–500.0) ^1^	266.6 (85.8–927.0) ^1^
–24 weeks	355.8 (39.8–500.0)	135.7 (27.0–662.1) ^1^
Responders to therapy, *n* = 42		
–baseline	366.8 (76.9–500)	770.5 (190.7–2393.1), *n* = 34
–2 weeks	310.5 (66.4–424.8) ^1^	665.5 (264.8–1481.6)
–4 weeks	409.5 (101.7–500.0)	312.2 (81.2–925.5) ^1^
–8 weeks	500 (84.4–500.0) ^1^	266.7 (85.8–927.0) ^1^
–24 weeks	355.7 (39.8–500.0)	134.7 (27.0–662.1) ^1^
Good response, *n* = 35		
–baseline	200.8 (51.5–500.0)	762.3 (106.9–2393.1), *n* = 29
–2 weeks	246.8 (62.6–436.0)	709.1 (116.6–1481.6)
–4 weeks	303.2 (57.2–500.0)	219.7 (75.2–960.3) 1
–8 weeks	252.6 (44.7–500.0)	233.3 (57.3–853.3) 1
–24 weeks	240.6 (19.5–500.0)	130.8 (17.3–624.9) 1
Moderate/no response, *n* = 7		
–baseline	403.2 (295.7–500.0)	803.7 (598.7–1233.4),
–2 weeks	364.1 (340.1–389.1)	627.9 (550.6–1623.5)
–4 weeks	500.0 (300.5–500.0) ^2^	462.0 (313.5–890.2)
–8 weeks	500.0 (500.0–500.0) ^2^	300.0 (191.6–965.4) ^1^
–24 weeks	500.0 (297.0–500.0)	464.4 (42.1–1000.0) ^1^

^1^*p* < 0.05 compared to baseline,  ^2^*p* < 0.05 between groups good effect and moderate effect.

The concentration of anti-CCP remained high during the entire period of TCZ therapy both in the group in general and in the patients who responded to therapy; 5% of anti-CCP-positive patients seroconverted to anti-CCP-negative results. The level of anti-MCV significantly decreased by 70, 69, and 82% after 4, 8, and 24 weeks, respectively, after the start of TCZ in the patients with a good response and by 62 and 42% of the baseline after 8 and 24 weeks, respectively, in the patients with a satisfactory response to therapy.

Given that the use of TCZ was accompanied by negative anti-CCP seroconversion, we estimated the percentage of patients who achieved immunological remission during treatment. By the 24th week of therapy, clinical remission and anti-CCP seroconversion was reached by three (7.1%) patients.

In this work, we obtained data on different values of anti-CCP and anti-MCV in RA. The level of anti-MCV positively correlated with the disease activity and was associated with the development of more pronounced destructive changes in the joints, in contrast to anti-CCP, the correlation of which with the disease activity indices, as well as with the level of acute-phase indices, was not found. Therapy also had a different effect on the level of antibodies. When using RTM, the concentration of anti-CCP remained high throughout the course of therapy, whereas the level of anti-MCV significantly decreased by the 12th and 24th weeks of observation. There are published data similar to ours on the absence of anti-CCP dynamics during RTM therapy [17, 18]; however, the authors point to a significant decrease in the concentration of anti-MCV in the blood sera of RA patients treated with RTM [19]. In the group of patients treated with TCZ, no statistically significant change in the level of anti-CCP was observed, whereas the concentration of anti-MCV significantly decreased from the 4th to the 24th week of treatment. Similar data were obtained by Sato et al. [20]. These authors found a significant decrease in the IgM RF content at the 12th and 52nd weeks of therapy, whereas the level of anti-CCP remained high throughout the treatment period, and three patients with low-positive anti-CCP titers showed seroconversion to the anti-CCP-negative variant.

It is believed that a pronounced decrease in the concentration of anti-MCV in RA patients who received DMARDs and biologics may be due to a greater dependence of these parameters on the inflammatory activity of the pathological process as compared to anti-CCP [21–23]. A correlation between an increase in the concentration of anti-MCV in blood and the clinical and laboratory activity of RA was found [24, 25]. In particular, in the study by Bang et al. [24] performed in a small sample of patients, direct correlation between the level of anti-MCV and DAS 28 was shown (*r* = 0.404). However, other researchers found no clear correlation between the inflammatory activity of the disease and the levels of anti-MCV in the blood serum of RA patients [26, 27]. In our work, a positive correlation between the levels of anti-MCV and the disease activity indices, as well as the number of swollen and painful joints, was found.

According to numerous studies, anti-CCPs are a more specific and stable serological marker of RA. They do not undergo seroconversion and are less dependent on the clinical and laboratory activity of the disease [21]. Against the background of therapy with biologics, the level of anti-CCP does not change or slightly decreases [18, 28]. According to our data, negative seroconversion of anti-CCP-positive results was observed in 7% of patients receiving the original RTM, in 15% of patients receiving a biosimilar, and in 5% of patients in the TCZ therapy group (mainly among the patients with initially low positive levels of these antibodies). Of interest are the data by Wunderlich et al. [29], who analyzed the effect of various DMARDs (MT monotherapy, TNF-α inhibitors, RTM, TCZ, and abatacept (ABC)) on the dynamics of anti-CCP levels in patients with RA. For 2.5 years of therapy, the authors revealed a significant decrease in the content of anti-CCP in the groups of patients who received RTM and ABC. This trend was more pronounced among the patients who responded well to treatment. Negative anti-CCP seroconversion was observed in five patients in the ABC group and two in the RTM group.

The contribution of ACPAs to the pathogenesis of RA is associated with a number of mechanisms: increased netosis (NETosis, Neutrophil Extracellular Trap) of neutrophils (a specific type of cell death with the formation of extracellular traps consisting of chromatin and granules capable of binding and destroying microorganisms), mediated by ACPAs; the severity of this process is correlated with hyperproduction of ACPAs and inflammatory mediators (pro-inflammatory cytokines, chemokines, and adhesion molecules) [30]. ACPAs are involved in the induction of osteoclastogenesis and bone resorption [31, 32]. It was established that ACPAs, interacting with vimentin on the membrane of osteoclast progenitors (OPs), are able to induce OP differentiation and thereby stimulate bone resorption. Recently, it was shown that polyclonal ACPAs, isolated from the sera of RA patients, induce osteoclastogenesis mediated by the chemokine IL-8 (CXCL1 in mice) [33], which is considered as the main effector molecule synthesized by OPs activated by ACPAs [34]. According to experimental studies, along with the induction of osteoclastogenesis, ACPAs are able to cause pain (mechanical and thermal hypersensitivity) in the absence of signs of inflammation [35]. The development of pain is also mediated by IL-8-dependent mechanisms, namely, by the binding of IL-8 (or CXCL1) to type 1 and 2 CXC chemokine receptors, which leads to sensitization and activation of sensory neurons [36].

In our study, we revealed the correlation between the hyperproduction of ACPAs and the development of destructive joint damage in RA patients. A high level of anti-MCV was more associated with radiographic indices of bone and cartilage tissue destruction (higher total Sharp score, as well as a greater number of joint space narrowing) as compared to anti-CCP. Similar data were obtained by a number of authors [37–40]. In the group of patients positive for anti-MCV, a higher frequency of radiological progression (according to the total Sharp score and Larsen index) and a greater number of erosions compared to anti-CCP-positive patients was found.

High levels of IgM RF and anti-CCP were significantly more frequently recorded among the patients high-positive for anti-MCV, which undoubtedly had an additional effect on the rate and severity of articular destruction in this group of patients. When analyzing the groups of patients high-positive and negative or low-positive for anti-CCP, we found no significant differences in the total Sharp score, as well as in the number of erosions and joint space narrowings in our group of patients (*p* > 0.05). Possibly, the cause for such differences is the different origin and epitope specificity of ACPA. Vimentin is a naturally occurring citrullinated protein that is synthesized and modified in synovial macrophages under the influence of proinflammatory cytokines. In contrast to synthetic cyclic citrullinated peptide 2, which has one citrullinated epitope, the modified citrullinated vimentin has a much larger number of epitopes (approximately 45) capable of binding to antibodies [8].

Thus, the data obtained in this study allow anti-MCV to be considered as a promising laboratory marker for identifying a group of patients with a potentially more severe course of the disease. The anti-MCV level correlates with inflammatory activity and development of bone tissue destruction and decreases during therapy. Anti-CCP is a more stable index and is important for diagnosing the disease; its level slightly changes during therapy and does not require monitoring.
