# Erratum to: **Different Clinical Relevance of Anti-Citrullinated Protein Antibodies in RA Patients**

**DOI:** 10.1134/S1607672923050034

**Published:** 2024-01-24

**Authors:** A. S. Avdeeva, M. V. Cherkasova, E. L. Nasonov

**Affiliations:** 1 Nasonova Research Institute of Rheumatology, Moscow, Russia; 2 Department of Rheumatology, Institute of Professional Education, Sechenov First Moscow State Medical University (Sechenov University), Moscow, Russia

The article “Different Clinical Relevance of Anti-Citrullinated Protein Antibodies in RA Patients”, written by A.S. Avdeeva, M.V. Cherkasova, and E.L. Nasonov, was originally published electronically in Springer-Link on October 13, 2023 without Open Access. After publication in volume 511, pages 87–194, the authors decided to make the article an Open Access publication. Therefore, the copyright of the article has been changed to © The Author(s) 2023 and the article is forthwith distributed under the terms of a Creative Commons Attribution 4.0 International License (http://creativecommons.org/licenses/by/4.0/, CC BY), which permits use, duplication, adaptation, distribution and reproduction of a work in any medium or format, as long as you cite the original author(s) and publication source, provide a link to the Creative Commons license, and indicate if changes were made.

